# Supplementary material for: Incidence of Carassius auratus Gibelio Gill Hemorrhagic Disease Caused by CyHV-2 Infection Can Be Reduced by Vaccination with Polyhedra Incorporating Antigens
Source: Vaccines (Basel). 2021 Apr 16;9(4):397. doi: 10.3390/vaccines9040397 (PMC8072653; doi:10.3390/vaccines9040397)
Supplement: Supplementary file 1 [file vaccines-09-00397-s001.zip › vaccines-1151515-supplementary.pdf]

Supplementary Materials:

The following are available online at www.mdpi.com/xxx/s1,Figure S1 H1-D4ORFs and D4ORFs-VP3 fused sequences**:** A, H1-D4ORFs sequence; B, D4ORFs-VP3 sequence. The sequence with the wavy line represents the corresponding sequence of region 1–186 nt of the *CyHV-2 ORF72* gene; the sequence with boldface represents the corresponding sequence of region 993–1197 nt of the *CyHV-2 ORF66* gene; the sequence with a double underline represents the corresponding sequence of region 603–783 nt of the *CyHV-2 ORF81* gene; the sequence in italics represents the corresponding sequence of region 85–186 nt of the *CyHV-2 ORF82* gene; the sequence in lowercase font represents the sequence of region 1–90 nt (H1-tag) of the BmCPV polyhedrin H1-helix in the H1-D4ORFs sequence; and the sequence in lowercase font with a single underline represents the sequence of region 1–279 nt (VP3 tag) of the *BmCPV VP3* gene in the D4ORFs-VP3 sequence. The sequences from CyHV-2 were optimized according to the preference of the silkworm BmNPV codons.

Figure S2 Antibody titer in the immunized fish**:**Serum samples (n = 3) harvested from the caudal vein of fish at 3 weeks post-vaccination with BmNPV-H1-D4ORF-polh or BmNPV-D4ORF-VP3-polh were stored overnight at 4 °C. After centrifugation for 10 min at 2,000 × g, the supernatants were collected and antibody titers in the sera were detected using an uncompetitive ELISA. ***, $p \leq 0.0001$.

Figure S3 and S4 were the original western blotting figures.

A

ATGgcagacgtagcaggaacaagtaaccgagactttcgcggacgcgaacaaagactattcaacagcgaacaata
caactataacaacagcATGTACGGTCTGAACAACGCTCAGGGTTTCATCGATACCGAGTGGATCGACAGACAGT
CAATCGCTATGACCGCTCAGGAAACAAGCAGACTGTTGAACCCGTACCTGGCTACAAAAGGTCAGAGAGTGGAC
CCTTCAAACCTGTACATCCCAGACGGTATCTTCGTGACATACACACCTACCGGT**TCAAGACACCCTATGGTGGA**
ATACGAACGCATCGAATCACAACTGGAACCAGACACAGGAGCTTCAAGCTTCGTGGACAAACTGGTGGAAAAGT
CAGACCTGTCAGCTTGCTTGTCACACACAGACGGTACAATCGAACTGCCTTCATCTTGGGTGGACCCTAGAGCT
AAACAGTTCCACGATCTGGACGACCTGATGATCTACGGTTCAGCAGATTACATGGACTCAGACGACGAAGATTA
CGATTCATACGCCGGAGCAGCAGAAACACTGCAAAGAAGCATGCGCGACTACGCTGACGAAGAAGATAGCGACA
TGGACGATAGCACATCACTGCTGAACAGCGTGAGAAAGATGAGCAGCAAGTTCAAGAGCACCGTGTAC*GAAGAC*
GACCAAACACAGAGCAGCAACACAACAGCTACAAGCGACCGCTACAAGTACTACACAAGCAGAGGTACAATCCA
*ACGCGCTAACAGACCGGGTTTG*TAA

B

ATGTACGGTCTGAACAACGCTCAGGGTTTCATCGATACCGAGTGGATCGACAGACAGTCAATCGCTATGACCGC
TCAGGAAACAAGCAGACTGTTGAACCCGTACCTGGCTACAAAAGGTCAGAGAGTGGACCCTTCAAACCTGTACA
TCCCAGACGGTATCTTCGTGACATACACACCTACCGG**TTCAAGACACCCTATGGTGGAATACGAACGCATCGAA**
TCACAACTGGAACCAGACACAGGAGCTTCAAGCTTCGTGGACAAACTGGTGGAAAAGTCAGACCTGTCAGCTTG
CTTGTCACACACAGACGGTACAATCGAACTGCCTTCATCTTGGGTGGACCCTAGAGCTAAACAGTTCCACGATC
TGGACGACCTGATGATCTACGGTTCAGCAGATTACATGGACTCAGACGACGAAGATTACGATTCATACGCCGGA
GCAGCAGAAACACTGCAAAGAAGCATGCGCGACTACGCTGACGAAGAAGATAGCGACATGGACGATAGCACATC
ACTGCTGAACAGCGTGAGAAAGATGAGCAGCAAGTTCAAGAGCACCGTGTAC*GAAGACGACCAAACACAGAGCA*
GCAACACAACAGCTACAAGCGACCGCTACAAGTACTACACAAGCAGAGGTACAATCCAACGCGCTAACAGACCG
*GGTTTG*atgtggcactatacgagtatcaacaatgacacgagagtagcacttgaccccaaaccaaaccaaattag
aacgataacaaaaccaaacacagtacctcaactcggcacagactacttgtacactttcaactcacaacgacgat
cacacacgttacgactattaggaccttttcagtacttcaactcttccgagacagatagaggacatccactattt
cgcttaccccttaagtatccgtcaaaagcaataccagcggatgagttaattgataacttgcatTAA

Figure S1. Synthetic D4ORF fusion sequence

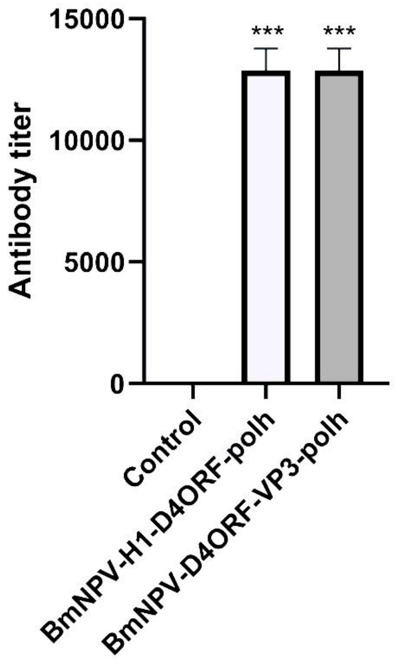

Figure S2. Valence of Antibody.

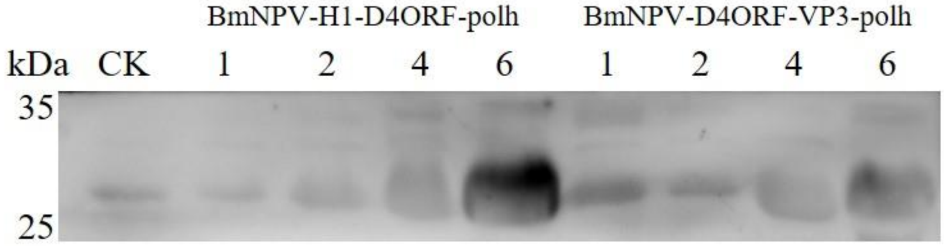

Figure S3. Western blot analysis depicting the hemolymph of silkworm larvae infected with BmNPV-H1-D4ORF-polh/BmNPV-D4ORF-VP3-polh.

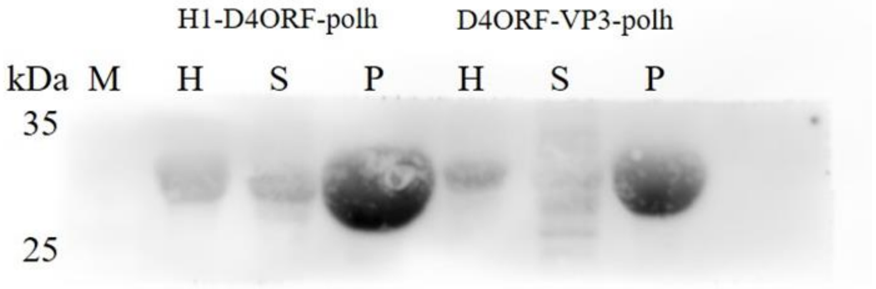

Figure S4. Western blot analysis of the hemolymph, supernatant of the hemolymph, and purified recombinant cytoplasmic polyhedra.
